# Comparable 5‐Year Survival in People With and Without HIV Following Hepatocellular Carcinoma Diagnosis: A Multicenter Study

**DOI:** 10.1111/liv.70437

**Published:** 2025-11-18

**Authors:** Massimo Iavarone, Eleonora Alimenti, Massimo Puoti, Hamid Hasson, Bianca Monti, Sherrie Bhoori, Francesco Peracchi, Alessia Siribelli, Marco Fava, Mariangela Bruccoleri, Marco Merli, Giulia Morsica, Annalisa De Silvestri, Paolo Bonfanti, Antonella Castagna, Alessandro Soria, Pietro Lampertico

**Affiliations:** ^1^ Foundation IRCCS Ca’ Granda Ospedale Maggiore Policlinico, Division of Gastroenterology and Hepatology Milan Italy; ^2^ CRC ‘A. M. and A. Migliavacca’ Center for Liver Disease, Department of Pathophysiology and Transplantation University of Milan Milan Italy; ^3^ School of Medicine University of Milano‐Bicocca Milan Italy; ^4^ ASST GOM Niguarda, Division of Infectious Diseases Milan Italy; ^5^ Unit of Infectious Diseases, IRCCS San Raffaele Scientific Institute Milan Italy; ^6^ Division of HPB Surgery, Hepatology and Liver Transplantation Fondazione IRCCS Istituto Nazionale Tumori Milan Italy; ^7^ Vita‐Salute San Raffaele University Milan Italy; ^8^ Clinic of Infectious Diseases, Fondazione IRCCS San Gerardo Dei Tintori Monza Italy; ^9^ Biometry and Clinical Epidemiology Scientific Direction IRCCS Policlinico San Matteo Foundation Pavia Italy

**Keywords:** AIDS, ART, BCLC, HCC, HIV, HIV‐negative overall survival, liver transplantation, surgery

## Abstract

**Background and Aims:**

Conflicting data on hepatocellular carcinoma (HCC) survival in people with HIV (PWH) may depend on tumour aggressiveness and access to curative treatments. We compared outcomes of two HCC cohorts according to HIV status.

**Methods:**

Patients from four tertiary referral centers in Northern Italy with the first HCC diagnosis (2005–2023) were analysed. Clinical characteristics, treatment access, and overall survival (OS) were described by HIV status, using inverse probability treatment weighting and propensity score (IPTW/PS) methods. Cox regression models assessed mortality predictors and recurrence after initial treatment.

**Results:**

Among 606 patients, 143 were PWH and 463 HIV‐negative. PWH were younger (median age 53 vs. 68 years, *p* < 0.001), predominantly male (87% vs. 75%, *p* = 0.004), with a lower proportion of Child‐Pugh A cirrhosis (71% vs. 76%, *p* = 0.01). Despite similar surveillance rates (91% vs. 88%), PWH more frequently presented with BCLC‐C stage HCC (22% vs. 10%, *p* < 0.001). First‐line curative treatments were offered to 47% of PWH and 60% of HIV‐negative patients (*p* = 0.006), while second‐line treatment rates were similar (42% vs. 44%, *p* = 0.91). During median follow‐up of 32 (11–78) and 36 (19–84) months for PWH and HIV‐negative subjects (*p* = 0.04), five‐year OS was 43.4% versus 44.1% (*p* = 0.97), median OS was 48 [interquartile range (IQR) 15–127] versus 46 (IQR 21–109) months (*p* = 0.97). Multivariable analysis after IPTW/PS showed similar mortality (HR 1.03, 95% CI 0.52–1.99, *p* = 0.94) but a higher two‐year recurrence rate in PWH (47.4% vs. 22.8%, *p* = 0.03).

**Conclusion:**

Despite more advanced tumours and higher recurrence, survival in PWH with HCC was similar. Access to curative treatments with aggressive downstaging strategies may counterbalance initial disadvantages.

**Abbreviations**
AFP: Alpha‐fetoprotein
AIDS: Acquired Immunodeficiency Syndrome
ALBI: Albumin‐bilirubin Grade for HCC
ALD: Alcoholic Liver Disease
ART: Antiretroviral Therapy
BCLC: Barcelona Clinic Liver Cancer
BMI: Body Mass Index
BSC: Best Supportive Care
CD4: CD4+ lymphocyte T‐cells (a type of white blood cell)
CI: Confidence Interval
CPT: Child‐Pugh Turcotte Score
CT: Computed Tomography
E/G: Oesophagogastric (oesophageal and/or gastric)
EHS: Extrahepatic Spread
HBV: Hepatitis B Virus
HCC: Hepatocellular Carcinoma
HCV: Hepatitis C Virus
HDV: Hepatitis Delta Virus
HE: Hepatic Encephalopathy
HIV: Human Immunodeficiency Virus
HR: Hazard Ratio
INR: International Normalised Ratio
IO: Immunotherapy
IPTW/PS: Inverse Probability of Treatment Weighting (IPTW)–propensity score approach
IQR: Interquartile Range
LT: Liver Transplantation
MASLD: Metabolic‐associated steatotic liver disease
MC: Milan Criteria
MELD: Model for End‐Stage Liver Disease
MRI: Magnetic Resonance Imaging
MVI: Macrovascular Invasion
NLR: Neutrophil/Lymphocyte Ratio
OS: Overall Survival
PWH: People with HIV
TA: Thermal Ablation
TACE: Transarterial Chemoembolization
TARE: Transarterial Radioembolization
TKI: Tyrosine Kinase Inhibitors
TTR: Time to Recurrence

**Summary**

- The Study provides a detailed account of treatment approaches and outcomes in one of the largest cohorts of PWH with HCC to date, covering an extended follow‐up period, encompassing initial and subsequent therapies, addressing a gap in previous research.
- Despite more advanced HCC at diagnosis, 5‐year survival in PWH was comparable to PWOH, following an optimised HIV management and equitable access to curative HCC therapies, including early LT.

## 1 Introduction

The introduction of combination antiretroviral therapy (ART) in the late 90s led to a sharp decline in AIDS‐related morbidity and mortality among people with HIV (PWH) [1, 2, 3], shifting the disease burden toward non‐AIDS conditions such as liver disease and non‐AIDS‐defining cancers, including hepatocellular carcinoma (HCC) [4, 5, 6, 7]. In PWH co‐infected with HBV/HCV, HIV‐related immune dysfunction, gut microbiota dysbiosis, and additional liver‐damaging factors (e.g., alcohol use, metabolic syndrome) accelerate fibrogenesis and oncogenesis, leading to more aggressive HCC presentations [8, 9, 10, 11, 12]. These patients often exhibit higher tumour burden and advanced disease at diagnosis, limiting treatment options and exacerbating disparities in care access [6, 9, 13, 14, 15, 16].

Data on HCC treatment and outcomes in PWH remain scarce and inconsistent due to small study populations and historical disparities in survival and care. While some studies report similar access to curative treatments in PWH [14, 17, 18], others highlight a greater reliance on non‐curative therapies or best supportive care (BSC) [9, 16, 19]. Consequently, whether PWH with HCC face reduced survival remains unresolved, with conflicting findings on mortality risks [6, 9, 14, 18, 19, 20, 21, 22, 23]. However, available evidence consistently indicates that treatment type strongly influences prognosis [9, 19, 21, 24], whereas HCC recurrence appears less impactful on survival, though studies remain limited by sample size and insufficient data on second‐line treatments [20, 22, 25].

Given the gaps in understanding access to care and outcomes in PWH with HCC, we conducted a study on patients treated since 2005—an era marked by widespread ART use and expanded transplant eligibility for PWH. Across four network hospitals offering equal care opportunities, we assessed clinical presentation, treatment access, and outcomes in PWH with newly diagnosed HCC, comparing them to a calendar‐matched cohort of people without HIV (PWOH).

## 2 Methods

This multicenter, retrospective cohort study included patients with a first HCC diagnosis from January 2005—when liver transplantation (LT) became available for PWH in our region—through March 2023. Patients were managed at four tertiary hospitals in Northern Italy (Lombardy): three centers enrolled PWH (PWH group), while the fourth enrolled PWOH (PWOH‐group). Each center provided comprehensive HCC treatment on‐site or via a referral network.

All patients were prospectively followed, with data retrospectively collected from electronic medical reports. Data collection was independently managed by each center and centrally revised for accuracy. Eligibility required documented HIV serology (anti‐HIV antibodies) and a histological or clinical first diagnosis of HCC per international guidelines [26, 27].

The study was approved by the Ethical Committee ‘Lombardia 3’ (ID 4428_S_P) and conducted in accordance with the Helsinki Declaration (1975, revised 2008), local laws, and EU Regulation 2016/679 on data protection. Informed consent was waived per Italian law due to the study's retrospective design.

### 2.1 Study Endpoints

The primary endpoint was overall survival (OS) from HCC diagnosis in both groups. Secondary endpoints included comparisons of clinical and demographic characteristics, liver disease stage at HCC diagnosis, treatment allocation, HCC recurrence rates and management, and predictors of OS and recurrence in PWH and the entire cohort.

### 2.2 Liver Disease Aetiology and Assessment

Liver disease aetiology was classified as viral if patients had HCV infection (detectable HCV RNA) and/or chronic HBV infection (HBsAg or detectable HBV DNA), with or without HDV coinfection. All patients with viral infections received antiviral therapy per national and international guidelines. Non‐viral etiologies included alcoholic liver disease (ALD; alcohol consumption > 20 g/day in females, > 30 g/day in males), metabolic‐associated steatotic liver disease (MASLD; steatosis with at least one cardiometabolic criterium among obesity, diabetes/pre‐diabetes, arterial hypertension, hypertriglyceridemia or low high‐density lipoprotein cholesterol in plasma), autoimmune hepatitis, and primary biliary cholangitis. Mixed aetiology was assigned when viral and non‐viral causes coexisted, while cryptogenic liver disease was diagnosed when all causes were excluded.

Cirrhosis was diagnosed histologically or non‐invasively (by liver stiffness > 15 kPa by elastography or clinically with indirect signs of advanced liver disease, such as thrombocytopenia [platelet count < 150 000 × 10^9^/L] associated with radiological signs of cirrhosis or portal hypertension, or varices) [28]. Cirrhosis severity was assessed using the Child‐Pugh‐Turcotte (CPT) score, liver function with the model for end‐stage liver disease (MELD) [29] and albumin‐bilirubin grade (ALBI) [30] scores, and clinically significant portal hypertension was defined per Baveno consensus criteria [28]. Patients were considered under HCC surveillance if they underwent biannual abdominal ultrasound screenings.

Baseline characteristics at HCC diagnosis included demographics, comorbidities, oesophageal/gastric varices (from the last gastroscopy), and laboratory data.

### 2.3 HIV History, Staging, and Treatment

The PWH group included patients with a prior HIV diagnosis. Collected variables included plasma HIV RNA levels, ART type and initiation date, prior AIDS‐defining events, CD4+ T‐cell count at enrollment, and CD4+ nadir since diagnosis.

### 2.4 HCC Diagnosis, Staging, and Treatment

HCC was diagnosed via contrast‐enhanced computed tomography (CT) or magnetic resonance imaging (MR) or histology, following international guidelines. Staging was based on the Barcelona Clinic Liver Cancer (BCLC) system [31]. Treatment decisions were made per center‐specific clinical practice, international guidelines, and multidisciplinary evaluation.

Treatment response was assessed with CT/MR 1 month post‐treatment, then every 3–4 months for 2 years or until recurrence, after which patients resumed six‐monthly surveillance unless otherwise indicated. Response to treatment was defined according to mRECIST criteria as: complete response if no viable tumour was observed at contrast‐enhanced imaging (no intratumoral contrast enhancement); partial response in patients with reduction ≥ 30% of viable tissue within the target lesion; progressive disease if an increase ≥ 20% of viable nodules was observed; stable disease for patients that did not match the criteria listed above. Data collected included HCC characteristics at diagnosis, treatment allocation for each line, first‐line treatment response, and recurrence. Curative treatments included liver transplantation, surgical resection, and radiofrequency/microwave thermoablation (TA).

### 2.5 Statistical Analysis

Continuous variables were reported as median and interquartile range (IQR) and compared using the Kruskal–Wallis one‐way analysis of variance, while categorical variables were expressed as counts (percentages) and analysed using Fisher's exact or Chi‐Square tests. Death and HCC recurrence after first‐line treatment were reported as percentages. Follow‐up began at HCC diagnosis and continued until death or last hospital visit. OS and OS rates were estimated using Kaplan–Meier analysis, while time to HCC recurrence and recurrence rates were calculated from first treatment to recurrence, death, or last visit, whichever occurred first. OS and recurrence were compared between PWH and PWOH using the log‐rank test. Analyses were conducted directly and after balancing clinical variables using Inverse Probability of Treatment Weighting (IPTW)/propensity (PS) score to adjust for confounders, including age, sex, body mass index (BMI), aetiology, cirrhosis, CPT, creatinine, platelet count, alpha‐fetoprotein (AFP), BCLC stage, and presence of extrahepatic spread (EHS) or macrovascular invasion (MVI) in a logistic model. We used the inverse of propensity score (1/PS) as a weight in the Cox proportional model to render subpopulations of individuals with and without HIV comparable. The analyses are done both for the entire sample and in the common support sample composed of all PWH plus those PWOH in the region of common support. Test of balance was made with an overidentification test. It tests whether the model‐adjusted means of the covariates are the same between groups. Cox univariate models were used to identify predictors of mortality and HCC recurrence in the full cohort and stratified by HIV status. Other than baseline variables, first‐line treatment was included in the analysis as a time‐varying variable. In PWH, additional factors (CD4+ T‐cell count, nadir CD4+, AIDS‐defining events, and HIV RNA > 1000 copies/mL) were tested. The proportional effect of all variables along time was preliminarily evaluated with Schoenfeld Residuals and Log–Log plots. The variables significant in univariable analysis were included in multivariable models, ensuring no collinearity. Multiple multivariable analyses were performed, including models with interaction between clinically related variables, selecting those demonstrating the best performances.

Finally, the Cox univariate model was used to test HIV as a predictor of mortality and HCC recurrence after IPTW/PS.

Statistical significance was set at *p* < 0.05, with results reported as HR and 95% Confidence Intervals (CI). Data analysis was performed using STATA 17.0 (Stata Corp, College Station, TX, USA).

## 3 Results

### 3.1 Patients' Baseline Characteristics

**FIGURE 1:**
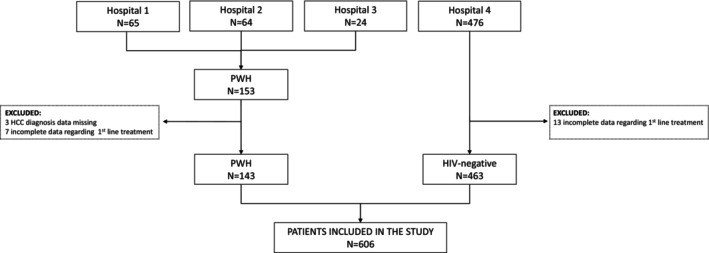
Patients' disposition.

From January 18, 2005, to March 12, 2024, 629 patients were diagnosed with HCC (153 PWH, 476 HIV‐negative). After excluding 23 patients (10 PWH, 13 PWOH) due to missing data, 606 remained (143 PWH, 463 PWOH) (Figure 1).

Patient and HCC characteristics at diagnosis are shown in Table 1. PWH were younger (median age 53 [49–57] vs. 68 [60–73] years, *p* < 0.001), more often male (87% vs. 75%, *p* = 0.004), and had a lower BMI (23.6 [21.0–26.7] vs. 25.3 [23.3–27.7], *p* < 0.001). Liver disease was primarily viral in PWH (99% vs. 77%, *p* < 0.001), while alcohol misuse was less common (30% vs. 39%, *p* = 0.004). Cirrhosis rates were similar, though PWH had a slightly lower CPT‐A classification (71% vs. 76%, *p* = 0.01). Before 2010, diagnosis of cirrhosis was histological in 67% of patients, and clinical in other patients. After 2010, diagnosis of cirrhosis was made mainly through elastography (73%), while a minority of patients underwent liver biopsy (10%) or received a clinical diagnosis only (7%).

**TABLE 1:** Characteristics of the 606 patients enrolled in the study and of hepatocellular carcinoma (HCC) at diagnosis.

Characteristic	Whole cohort (*n* = 606)	PWH group (*n* = 143)	HIV‐negative group (*n* = 463)	*p* value
Age, years^a^	64 (55–72)	53 (49–57)	68 (60–73)	< 0.001
Males, *N* (%)	472 (78)	124 (87)	348 (75)	0.004
BMI, kg/m^2a^	25 (22.7–27.4)	23.6 (21.0–26.7)	25.3 (23.3–27.7)	< 0.001
Diabetes, *N* (%)	122/510 (24)	25/126 (20)	97/384 (25)	0.21
Alcohol misuse, *N* (%)	213/582 (37)	30/119 (30)	183 (39)	0.004
Aetiology, *N* (%)				< 0.001
HCV	309 (62)	93 (65)	286 (62)	
HBV	54 (9)	9 (6)	45 (10)	
HCV + HBV	46 (8)	32 (22)	14 (3)	
HDV	19 (3)	8 (6)	11 (2)	
Non‐viral	108 (18)	1 (1)	107 (23)	
Diagnosis of cirrhosis, *N* (%)	576 (95)	141 (99)	437 (94)	0.04
CPT, *N* (%)				
A	423/565 (73)	91/128 (71)	332/437 (76)	0.01
B	123/565 (22)	27/128 (21)	96/437 (22)	
C	19/565 (3)	10/128 (8)	9/437 (2)	
MELD^a^	9 (7–11)	9 (7–12)	9 (7–10)	0.41
ALBI grade, *N* (%)				0.12
1	264/566 (47)	45/114 (39)	219/452 (49)	
2	287/566 (51)	64/114 (56)	223/452 (49)	
3	15/566 (2)	5/114 (4)	10/452 (2)	
Ascites, *N* (%)	169/601 (28)	27/139 (19)	142 (30)	0.009
HE, *N* (%)	56/597 (9)	9/135 (7)	47/462 (10)	0.22
E/G varices, *N* (%)	203/574 (35)	44/130 (34)	159/444 (36)	0.68
Previous AIDS‐defining events, *N* (%)	NA	34/132 (26)	NA	
CD4 count, cells/mm^3a^	NA	414.5 (260–606)	NA	
CD4 count, *N* (%)	NA		NA	
< 200 cells/mm^3^		21/130 (16)		
≥ 200 cells/mm^3^		109/130 (84)		
CD4 nadir, cells/mm^3a^	NA	144 (81–250)	NA	
CD4 nadir	NA		NA	
< 200 cells/mm^3^		61/97 (63)		
200–500 cells/mm^3^		30/97 (31)		
> 500 cells/mm^3^		6/97 (6)		
HIV RNA‐positive, *N* (%)	NA	9/127 (7)	NA	
Albumin, g/dL^a^	3.9 (3.5–4.3)	3.9 (3.5–4.1)	4 (3.6–4.3)	0.07
Total bilirubin, mg/dL^a^	0.9 (0.7–1.4)	0.9 (0.7 1.6)	0.9 (0.7–1.3)	0.08
INR^a^	1.1 (1.0–1.2)	1.0 (1.1–1.4)	1.1 (1.0–1.2)	0.32
Creatinine, mg/dL^a^	0.9 (0.8–1.1)	1.0 (0.8–1.1)	0.9 (0.7–1.0)	< 0.001
Platelet count, 10^9^ cells/L^a^	119.5 (84–173)	110 (66–151.5)	128.5 (88–181)	0.002
NLR^a^	2.2 (1.4–3.2)	1.8 (1.2–2.4)	2.4 (2.0–3.5)	< 0.001
AFP, ng/mL^a^	13.8 (5.2–84.5)	27.8 (6.9–347.7)	13 (5–60)	0.003
AFP > 200 ng/mL, *N* (%)	100/572 (17)	33/117 (28)	67/455 (15)	0.001
Number of nodules, *N* (%)				0.60
1	346 (57)	79 (55)	267 (58)	
2–3	173 (29)	45 (32)	128 (27)	
> 3	86 (14)	18 (13)	68 (15)	
Maximum diameter, cm^a^	2.7 (2.0–4.0)	2.8 (2.0–3.8)	2.7 (2.0–4.0)	0.93
MVI and/or EHS, *N* (%)				< 0.001
None	520 (86)	106 (74)	414 (89)	
MVI	48 (8)	22 (15)	26 (6)	
EHS	16 (2)	3 (2)	13 (3)	
Both	22 (4)	12 (9)	10 (2)	
‘Milan criteria’ out, *N* (%)	226 (37)	62 (43)	164 (35)	0.09
BCLC, *N* (%)				< 0.001
0	118 (20)	21 (15)	97 (21)	
A	285 (47)	59 (41)	226 (49)	
B	98 (18)	16 (11)	82 (18)	
C	79 (11)	31 (22)	48 (10)	
D	26 (4)	16 (11)	10 (2)	

Abbreviations: AFP, alpha‐fetoprotein; AIDS, acquired immunodeficiency syndrome; ALBI score, albumin‐bilirubin score; BCLC, Barcelona Clinic Liver Cancer; BMI, body mass index; CD, cluster of differentiation; CPT, Child‐Pugh score; E/G: Oesophagogastric; EHS, extrahepatic spread; HBV, hepatitis B; HCV, hepatitis C; HDV, hepatitis delta; HE, hepatic encephalopathy; HIV, human immunodeficiency virus; INR, international normalised ratio; MELD, model for end‐stage liver disease; MVI, macrovascular invasion; NLR, neutrophil‐to‐lymphocyte ratio.

^a^ Median (IQR).

At HCC diagnosis, PWH had been on ART for a median of 14.5 years (IQR 1–34), with only 5 (3%) not receiving ART. HCC detection through six‐month surveillance was comparable (91% vs. 88%, *p* = 0.30). Tumour burden at presentation was similar, but PWH had a higher proportion of advanced BCLC‐C stage (22% vs. 10%, *p* < 0.001), more frequent MVI and/or EHS (27% vs. 11%, *p* < 0.001), and elevated AFP > 200 ng/mL (28% vs. 15%, *p* = 0.001). In patients with BCLC C HCC, the tumour burden was lower in PWH as compared to PWOH, without reaching statistical significance: median maximum nodule diameter was 32 (IQR 25–50) vs. 48 (IQR 26–87) mm (*p* = 0.06), the proportion of patients with more than 3 nodules was 19% versus 46% (*p* = 0.04) and a higher number of patients were classified as advanced stage for deteriorated ECOG PS (12% vs. 2%, *p* = 0.08).

Moreover, between PWH with CD4+ lymphocyte count above or below 200/mm^3^ no significant differences were observed in CPT class (CPT A 71% vs. 66%, respectively, *p* = 0.92) and HCC stage at diagnosis (BCLC 0/A 55% vs. 58%; BCLC B 11% vs. 14%; BCLC C 22% vs. 14%; BCLC D 13% vs. 14% respectively, *p* = 0.19).

Baseline characteristics after IPTW propensity score matching are reported in Table S1.

### 3.2 Overall Survival

The median (IQR) follow‐up was 32 (11–78) months for PWH and 36 (19–84) months for PWOH (*p* = 0.04). By May 31, 2024, 90 PWH (63%) and 342 PWOH (74%) had died (*p* = 0.01). Among PWH, 56 (69%) patients died due to HCC progression, 15 (19%) for underlying liver disease and 10 (12%) for non‐hepatic causes; among PWOH death was attributed to HCC progression in 251 (74%), underlying liver disease in 42 (12%) and 44 (13%) non‐hepatic causes. No significant differences in terms of causes of death were observed between the two cohorts (*p* = 0.36).

**FIGURE 2:**
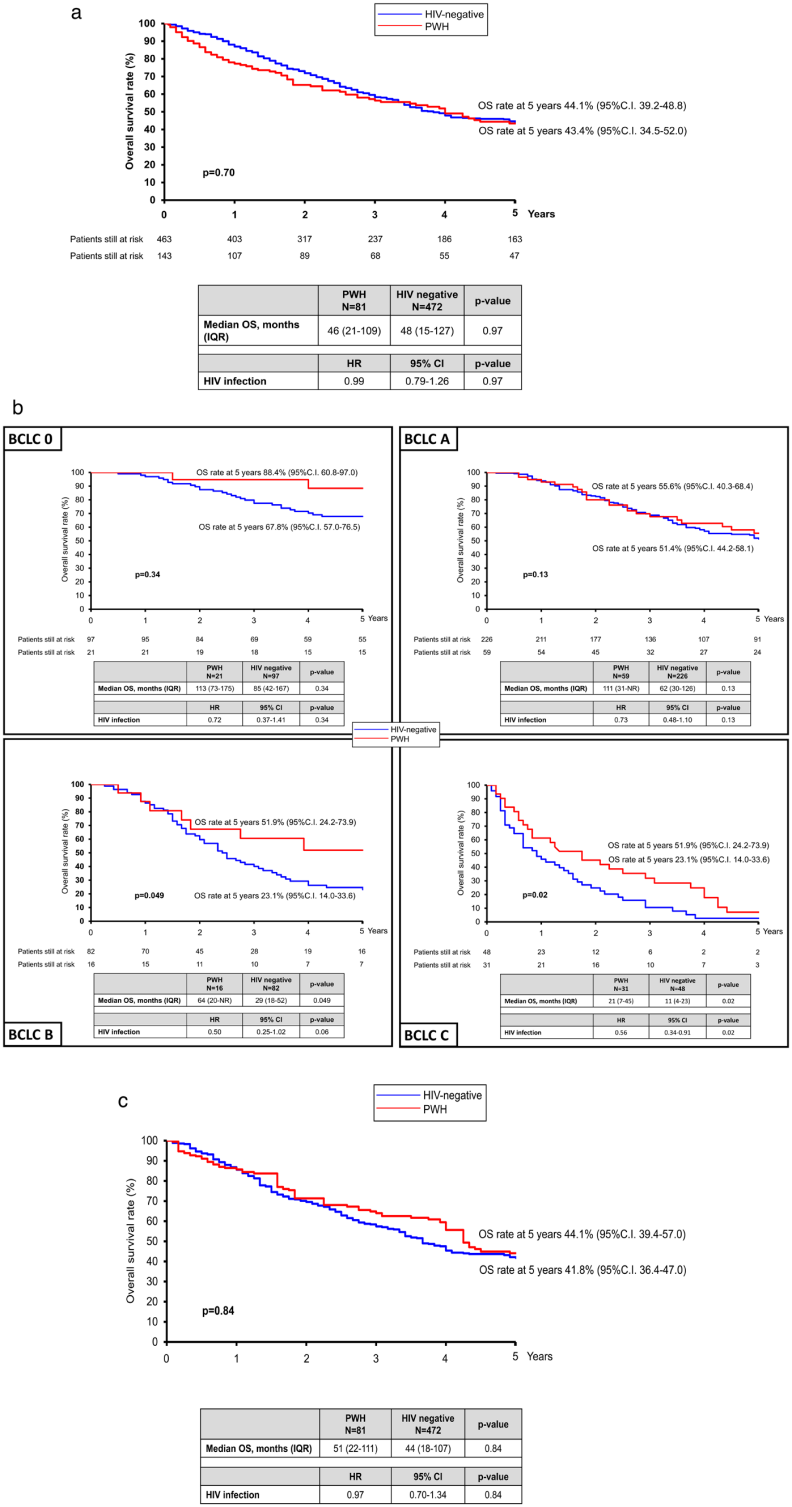
(a) Overall survival (OS) according to HIV status, direct comparison; (b) OS according to HIV status in each BCLC stage, direct comparison; (c) OS according to HIV status after clinical variable balancing with IPTW propensity score approach.

Median (IQR) OS was similar between groups (48 [15–127] vs. 46 [21–109] months, *p* = 0.97), with 2‐ and 5‐year survival rates of 65.2% (95% CI 56.6–72.6) and 43.4% (95% CI 34.5–52.0) in PWH vs. 71.9% (95% CI 67.5–75.8) and 44.1% (95% CI 39.2–48.8) in PWOH (*p* = 0.97) (Figure 2a).

Survival was comparable in BCLC 0 (median [IQR] 113 [73–175] vs. 85 [42–167] months, *p* = 0.34) and BCLC A (111 [31–not reached] vs. 62 [30–126] months, *p* = 0.13). However, PWH had significantly longer OS in BCLC B (64 [20–not reached] vs. 29 [18–52] months, *p* = 0.049) and BCLC C (21 [7–45] vs. 11 [4–23] months, *p* = 0.02) (Figure 2b).

After IPTW propensity score matching, OS remained similar (51 [22–111] vs. 44 [18–107] months, *p* = 0.84), with 2‐ and 5‐year survival rates of 71.3% (95% CI 59.0–80.6) vs. 69.5% (95% CI 64.5–74.0) and 44.1% (95% CI 39.4–57.0) vs. 41.8% (95% CI 36.4–47.0) (*p* = 0.84) (Figure 2c).

Considering only HCC progression as a cause of death, a similar survival was confirmed between PWH and PWOH in the whole population [median OS 91 (IQR 27‐NR) vs. 70 (IQR 26–197) months (*p* = 0.23)]. Similarly, no differences were observed in early and intermediate stages (BCLC 0 NR (IQR 76‐NR) vs. 152 (IQR 67‐NR) months, *p* = 0.35; BCLC A 127 (IQR 59‐NR) vs. 91 (IQR 40–197) months, *p* = 0.14; BCLC B 20 (IQR 13–22) vs. 28 (IQR 17–40) months, *p* = 0.09). On the other hand, it was confirmed the survival advantage of PWH as compared to PWOH in BCLC B and C [NR (IQR 21‐NR) vs. 32 (IQR 20–63) months, *p* = 0.01; 27 (IQR 10–51) vs. 12 (IQR 4–29) months, *p* = 0.003]. Lastly, in PWH, we observed that patients with a CD4+ lymphocyte count ≥ 200/mm^3^ had a longer OS as compared with patients with a lower count [51 (IQR 13–127) vs. 27 (IQR 20–37) months, *p* = 0.03]. Due to the low number of events, we did not perform an analysis for the single causes of death, but only for liver‐related (including both HCC progression and underlying liver disease), which confirmed the previous findings: patients with CD4 + ≥ 200/mm^3^ had a median OS of 88 (IQR 15‐NR) months vs. 27 (IQR 22–42) months in those with lower CD4+, *p* = 0.04.

### 3.3 Predictors of Mortality

In the entire cohort of 606 patients, variables significantly associated with mortality in the univariate analysis are reported in Table 2.

**TABLE 2:** Univariable and multivariable Cox regression model to predict mortality in the whole cohort (427 patients, 329 events).

Variable	Type of variable	HR	95% CI	*p* value	HR	95% CI	*p* value
Age, years	Continuous	1.02	1.01–1.03	< 0.001	1.02	1.01–1.03	< 0.001
Born males	Yes versus no	1.08	0.86–1.36	0.52			
BMI, kg/m^2^	Yes versus no	0.99	0.96–1.01	0.36			
Diabetes	Yes versus no	1.13	0.89–1.44	0.30			
HCV	Yes versus no	0.95	0.78–1.16	0.62			
HBV	Yes versus no	1.01	0.73–1.41	0.94			
HBV + HDV	Yes versus no	0.75	0.42–1.33	0.33			
HCV + HBV	Yes versus no	0.89	0.61–1.28	0.52			
Non‐viral	Yes versus no	1.23	0.96–1.58	0.11			
Cirrhosis	Yes versus no	1.47	0.86–2.50	0.16			
CPT							
A		1 (reference)			1 (reference)		
B	B versus A	1.59	1.26–2.00	< 0.001	1.17	0.89–1.54	0.25
C	C versus A	8.61	5.02–14.68	< 0.001	1.17	0.54–2.52	0.69
ALBI grade					Omitted for collinearity		
1		1 (reference)					
2	2 versus 1	1.15	0.94–2.00	0.16			
3	3 versus 1	1.84	1.00–3.89	0.049			
MELD	Continuous	1.06	1.03–1.10	< 0.001	Omitted for collinearity		
Ascites	Yes versus no	1.35	1.10–1.66	0.004	Omitted for collinearity		
HE	Yes versus no	1.60	1.17–2.18	0.003	Omitted for collinearity		
E/G Varices	Yes versus no	1.48	1.22–1.81	< 0.001	1.38	1.10–1.872	0.005
Albumin, g/dL	Continuous	0.73	0.61–0.86	0.001	Omitted for collinearity		
Bilirubin, mg/dL	Continuous	1.11	1.00–1.24	0.046	Omitted for collinearity		
INR	Continuous	1.62	1.26–2.07	< 0.001	Omitted for collinearity		
Creatinine, mg/dL	Continuous	1.64	1.17–2.31	0.004	1.13	0.80–1.92	0.19
Platelets, cells/mm^3^	Continuous	1.00	0.99–1.00	0.37			
NLR	Continuous	1.12	0.99–1.27	0.05			
AFP > 200 ng/mL	Yes versus no	2.58	2.03–3.29	< 0.001	1.85	1.39–2.46	< 0.001
Nodules categories					Omitted for collinearity		
1 nodule		1 (reference)					
2–3 nodules	2–3 nodules versus single	1.47	1.18–1.81	0.001			
> 3 nodules	> 3 nodules versus single	2.93	2.24–3.85	< 0.001			
Max diameter, cm	Continuous	1.01	1.01–1.02	< 0.001	Omitted for collinearity		
MVI, EHS or both					Omitted for collinearity		
None		1 (reference)					
MVI	Versus none	4.97	3.60–6.87	< 0.001			
EHS	Versus none	3.64	2.18–6.15	< 0.001			
Both	Versus none	6.77	4.31–10.62	< 0.001			
‘Milan criteria’ out	Yes versus no	2.80	2.30–3.40	< 0.001	Omitted for collinearity		
BCLC							
0		1 (reference)			1 (reference)		
A	Versus 0	1.41	1.08–1.86	0.013	1.45	1.06–1.97	0.02
B	Versus 0	2.58	1.86–3.58	< 0.001	2.20	1.48–3.27	< 0.001
C	Versus 0	6.97	4.97–9.78	< 0.001	4.07	2.56–6.47	< 0.001
D	Versus 0	17.61	10.80–28.71	< 0.001	6.75	3.39–13.42	< 0.001
First line treatment*							
LT		1 (reference)			1 (reference)		
Resection	Versus LT	1.90	1.02–3.54	0.04	1.22	0.51–2.91	0.66
TA	Versus LT	2.19	1.22–3.95	0.008	1.54	0.66–3.55	0.32
TACE	Versus LT	3.85	2.12–7.00	< 0.001	2.35	1.00–5.50	0.05
TARE	Versus LT	2.72	0.96–7.76	0.06	1.48	0.40–5.44	0.56
Systemic treatment	Versus LT	13.6	7.17–25.78	< 0.001	3.01	1.19–7.63	0.02
BSC only	Versus LT	30.9	15.55–61.30	< 0.001	7.12	2.74–18.54	< 0.001

Abbreviations: AFP, alpha‐fetoprotein; ALBI grade, albumin‐bilirubin score; BCLC, Barcelona Clinic Liver Cancer; BMI, body mass index; BSC, best supportive care; CI, confidence interval; CPT, Child‐Pugh score; E/G: Oesophagogastric; EHS, extrahepatic spread; HBV, hepatitis B; HCV, hepatitis C; HDV, hepatitis delta; HE, hepatic encephalopathy; HIV, human immunodeficiency virus; HR, hazard ratio; INR, international normalised ratio; LT, liver transplant; MELD, model for end‐stage liver disease; MVI, macrovascular invasion; NLR, neutrophil‐to‐lymphocyte ratio; TA, thermal ablation; TACE, transarterial chemoembolization; TARE, transarterial radioembolization.

* Upfront treatment.

In the multivariable analysis, older age, presence of oesophageal or gastric varices, AFP > 200 ng/mL and a more advanced BCLC stage were independent predictors of mortality, with HRs increasing progressively for stage from A to D.

Treatment choice also influenced mortality, with non‐curative treatments [TACE (HR 2.35, 95% CI 1.00–5.50, *p* = 0.05); systemic therapy (HR 3.01, 95% CI 1.19–7.64, *p* = 0.02); best supportive care (HR 7.12, 95% CI 2.74–18.54, *p* < 0.001)] showing higher mortality compared to liver transplantation (Table 2).

The impact of HIV infection appeared to be non‐proportional over time (log–log plot shown in Figure S1). For this reason, a piecewise analysis was performed and indicated that HIV infection was associated with an increased risk of death in the first 12 months after HCC diagnosis (HR 1.88, 95% CI 1.23–2.90, *p* = 0.004), not observed in later periods. However, this association was not confirmed after IPTW propensity score analysis, even in the first 12 months from HCC diagnosis (HR 1.03, 95% CI 0.52–1.99, *p* = 0.94). When considering only PWH, independent predictors of mortality included AFP > 200 ng/mL, intermediate, advanced or end‐stage HCC at diagnosis, and being a candidate for systemic treatment or best supportive care (Table 3). Independent predictors of mortality in PWOH are reported in Table S2.

**TABLE 3:** Univariable and multivariable Cox regression model to predict mortality in 97 PWH (65 events).

Variable	Type of variable	HR	95% CI	*p* value	HR	95% CI	*p* value
Age, years	Continuous	0.97	0.93–1.00	0.08			
Males	Yes versus no	0.75	0.41–1.35	0.33			
BMI	Continuous	1.00	0.94–1.06	0.96			
Diabetes	Yes versus no	1.12	0.64–1.94	0.69			
HCV	Yes versus no	1.11	0.72–1.72	0.64			
HBV	Yes versus no	0.99	0.40–2.45	0.98			
HBV + HDV	Yes versus no	0.54	0.20–1.49	0.23			
HCV + HBV	Yes versus no	1.00	0.61–1.65	0.99			
Non‐viral	Yes versus no	23.23	2.80–193.00	0.004	Omitted for collinearity		
CPT							
A		1 (reference)			1 (reference)		
B	Versus A	3.56	2.16–5.87	< 0.001	0.85	0.34–2.12	0.72
C	Versus A	9.03	3.55–22.96	< 0.001	0.52	0.12–2.18	0.37
ALBI grade							
1		1 (reference)					
2	Versus 1	0.95	0.60–1.51	0.84			
3	Versus 1	1.00	0.30–3.28	0.99			
MELD	Continuous	1.08	1.02–1.13	0.006	Omitted for collinearity		
NLR	Continuous	1.12	0.93–1.34	0.23			
Ascites	Yes versus no	1.98	1.20–3.28	0.008	Omitted for collinearity		
HE	Yes versus no	2.42	1.11–5.29	0.03	Omitted for collinearity		
E/G varices	Yes versus no	2.08	1.32–3.24	0.001	1.91	0.86–4.21	0.11
AIDS	Yes versus no	1.90	1.19–3.03	0.007	1.45	0.77–2.74	0.26
CD4 count							
≥ 200 cells/mm^3^		1 (reference)			1 (reference)		0.87
< 200 cells/mm^3^	Versus ≥ 200	1.81	1.04–3.17	0.04	1.08	0.46–2.53	
CD4 nadir							
> 500 cells/mm^3^		1 (reference)					
200–500 cells/mm^3^	Versus > 500	1.77	0.51–6.13	0.37			
< 200 cells/mm^3^	Versus > 500	1.55	0.47–5.14	0.47			
HIV RNA positive	Yes versus no	1.52	0.73–3.16	0.26			
Albumin, g/dL	Continuous	0.81	0.55–1.22	0.33			
Bilirubin, mg/dL	Continuous	1.02	0.84–1.25	0.82			
INR	Continuous	1.62	1.23–2.12	0.001	Omitted for collinearity		
Creatinine, mg/dL	Continuous	1.58	0.79–3.15	0.20			
Platelets, cells/mm^3^	Continuous	1.00	0.99–1.00	0.45			
AFP > 200 ng/mL	Yes versus no	2.53	1.59–4.03	< 0.001	3.54	1.92–6.52	< 0.001
‘Milan Criteria’ out	Yes versus no	3.72	2.40–5.77	< 0.001	Omitted for collinearity		
Nodules categories					Omitted for collinearity		
1 nodule		1 (reference)					
2–3 nodules	Versus single	1.40	0.88–2.23	0.15			
> 3 nodules	Versus single	2.62	1.42–4.84	0.002			
Maximum diameter, cm	Continuous	1.01	1.01–1.02	0.001	Omitted for collinearity		
MVI, EHS or both					Omitted for collinearity		
None							
MVI	Versus intrahepatic	4.78	2.77–8.26	< 0.001			
EHS	Versus intrahepatic	2.83	0.88–9.14	0.08			
Both	Versus intrahepatic	5.18	2.65–10.12	< 0.001			
BCLC							
0		1 (reference)			1 (reference)		
A	Versus 0	1.44	0.68–3.07	0.34	1.65	0.62–4.34	0.31
B	Versus 0	2.08	0.82–5.28	0.12	3.66	1.00–13.32	0.05
C	Versus 0	6.44	2.97–13.98	< 0.001	5.86	1.95–17.62	0.002
D	Versus 0	18.93	7.85–46.67	< 0.001	9.20	2.03–41.72	0.004
Treatment							
LT		1 (reference)			1 (reference)		
Resection	Versus LT	2.70	0.89–8.12	0.08	1.52	0.36–6.42	0.57
TA	Versus LT	2.68	0.90–7.98	0.08	9.81	0.24–3.97	0.98
TACE	Versus LT	2.35	0.79–6.96	0.12	9.35	0.21–4.02	0.83
TARE	Versus LT	1.55	0.28–8.54	0.62	4.42	0.04–4.98	0.51
Systemic treatment	Versus LT	19.88	6.04–65.39	< 0.001	5.09	1.04–24.98	0.04
BSC only	Versus LT	40.85	12.17–137.122	< 0.001	6.59	1.28–33.74	0.02

Abbreviations: AFP, alpha‐fetoprotein; AIDS, acquired immunodeficiency syndrome; ALBI grade, albumin‐bilirubin score; BCLC, Barcelona Clinic Liver Cancer; BMI, body mass index; BSC, best supportive care; CD, cluster of differentiation; CI, confidence interval; CPT, Child‐Pugh score; E/G: Oesophagogastric; EHS, extrahepatic spread; HBV, hepatitis B; HCV, hepatitis C; HDV, hepatitis delta; HE, hepatic encephalopathy; HIV, human immunodeficiency virus; HR, hazard ratio; INR, international normalised ratio; LT, liver transplant; MELD, model for end‐stage liver disease; MVI, macrovascular invasion; NLR, neutrophil/lymphocyte ratio; TA, thermal ablation; TACE, transarterial chemoembolization; TARE, transarterial radioembolization.

### 3.4 HCC Treatment and Recurrence

After HCC diagnosis, 69 (47%) PWH and 280 (60%) PWOH received curative treatments (*p* = 0.006). No significant difference was found in complete response to first‐line treatment: 56 (39%) in PWH vs. 206 (44%) in PWOH (*p* = 0.26, Table 4). When stratified by BCLC stage, PWH had lower access to curative treatment in BCLC A (35 [59%] vs. 171 [76%], *p* = 0.01), but more PWH with BCLC C received curative treatment compared to PWOH (12 [38%] vs. 4 [8%], *p* = 0.0029, Table S3). LT was more frequently offered to PWH (17% vs. 8%, *p* = 0.002).

**TABLE 4:** First and second‐line treatment allocation and response according to HIV status.

First line treatment			
Treatments	PWH (*n* = 143)	PWOH (*N* = 463)	*p* value
			< 0.001
LT	10 (7%)	16 (3.5%)	
Resection	32 (22%)	75 (16.2%)	
TA	27 (19%)	172 (37.1%)	
Resection + TA	0	5 (1.1%)	
TA + TACE	0	12 (2.6%)	
TACE	38 (26.6%)	112 (24.2%)	
TACE + TKI	1 (0.7%)	3 (0.6%)	
TARE	5 (4%)	5 (1.1%)	
Systemic treatment, TKI	12 (8%)	38 (8.2%)	
Systemic treatment, IO‐based	1 (0.7%)	4 (0.9%)	
BSC only	17 (12%)	21 (4.5%)	
Curative treatments^a^	69 (48%)	280 (60%)	0.01
Response to 1st line treatment			< 0.001
Complete response	56 (39%)	206 (44%)	
Partial response	33 (23%)	111 (24%)	
Stable disease	19 (13%)	8 (2%)	
Progression	35 (24%)	138 (30%)	

Second line treatment			
Treatments	PWH (*n* = 43)	PWOH (*n* = 115)	*p* value
			< 0.001
LT	3 (7%)	12 (10%)	
Resection	3 (7%)	3 (3%)	
TA	10 (23%)	33 (29%)	
Resection + TA	0	1 (1%)	
TA + TACE	0	1 (1%)	
TACE	8 (19%)	52 (45%)	
TACE + TKI	1 (2%)	0	
TARE	2 (4%)	1	
Systemic treatment, TKI	11 (26%)	8 (7%)	
Systemic treatment, IO‐based	0	1 (1%)	
BSC only	5 (12%)	3 (3%)	
Curative treatments at 2nd line^a^	16 (37%)	50 (43%)	0.48

Abbreviations: BSC, best supportive care; IO, immunotherapy; LT, liver transplantation; PWH, people with HIV; PWOH, people without HIV; TA, thermal ablation; TACE, transarterial chemoembolization; TARE, transarterial radioembolization; TKI, tyrosine kinase inhibitor.

^a^ Curative treatments included liver transplantation, surgical resection, and radiofrequency or microwave thermal ablation.

Among PWH, even if a trend toward easier access to curative treatment for patients with CD4+ lymphocyte count ≥ 200/mm^3^ was observed, the difference did not prove to be statistically significant (50% vs. 33%, *p* = 0.19).

**FIGURE 3:**
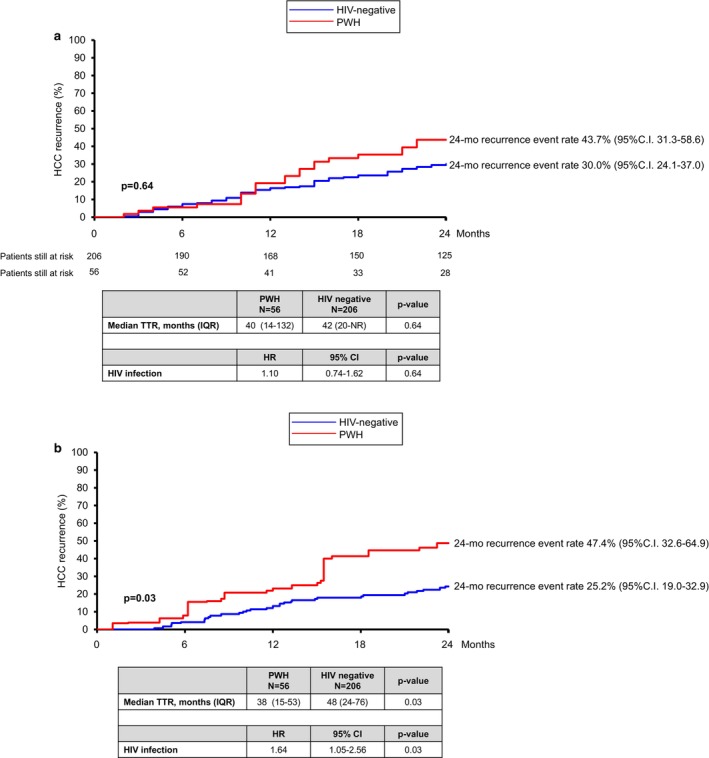
(a) HCC recurrence according to HIV status: Direct comparison; (b) HCC recurrence according to HIV status after clinical variable balancing with IPTW propensity score approach.

Among the 262 patients with complete response, HCC recurrence occurred in 33/56 (60%) PWH and 115/206 (56%) PWOH (*p* = 0.68). Median time to recurrence was 40 months (14–132) in PWH versus 42 months (20–not reached) in PWOH (*p* = 0.64). Recurrence rates at 1 and 2 years were 19.2% and 43.7% in PWH versus 16.4% and 30.0% in PWOH, respectively (Figure 3a). After IPTW propensity score adjustment, median time to recurrence was shorter in PWH (38 [15–53] months vs. 48 [24–76] months, *p* = 0.03), and the 2‐year recurrence rate was higher in PWH (47.4% [95% CI 32.6–64.9] vs. 25.2% [95% CI 19.0–32.9], *p* = 0.03) (Figure 3b).

Lastly, we compared HCC recurrence between PWH with CD4+ lymphocytes above or below 200/mm^3^. Median time to recurrence was longer in patients with higher CD4+ count [45 (14–112) vs. 16 (IQR 13–21) months] even if the difference was not statistically significant, due to the low number of patients (*p* = 0.05). The corresponding recurrence rate at 1 and 2 years was 21.2% (95% CI 11.2–38.0) and 37.8% (95% CI 24.3–55.4) in the group with CD4 + ≥ 200/mm^3^ versus 17% (95% CI 2.5–75.7) and 100% in the group with CD4 + < 200/mm^3^, respectively (*p* = 0.05).

Second‐line treatment was offered to 38/42 (90%) PWH and 112/115 (97%) PWOH after recurrence. Curative treatment was administered to 14/33 (42%) PWH and 50/115 (44%) PWOH (*p* = 0.91, Table 4).

Given the extended period of enrolment, patients were divided into three distinct cohorts according to the year of HCC diagnosis (Cohort 1 from 2005 to 2010; Cohort 2 from 2011 to 2015; Cohort 3 from 2016 to 2023) to estimate the evolution of treatment allocation over time. There were no significant differences in access to radical treatment between PWH and PWOH across the three periods (48% for Cohort 1 vs. 47% for Cohort 2 vs. 51% for Cohort 3, *p* = 0.89; and 62% for Cohort 1 vs. 54% for Cohort 2 vs. 57% for Cohort 3, *p* = 0.59, respectively). Despite the absence of statistical significance, an upward trend in the proportion of PWH undergoing LT as a primary treatment modality has been observed in recent decades (4% in Cohort 1 vs. 9% in Cohort 2 vs. 7% in Cohort 3, *p* = 0.70), also when access to LT at any point during the follow‐up period was considered: 10% in Cohort 1, 31% in Cohort 2, 21% in Cohort 3, *p* = 0.08 (Table S4).

### 3.5 Predictors of HCC Recurrence

Predictors of HCC recurrence after complete radiological response in the whole cohort (262 patients) by multivariate analysis included the presence of hepatic encephalopathy, higher serum creatinine, and HCC treatment different from LT (resection, TA and TACE) (Table S5).

However, after IPTW/PS, HIV appeared to be associated with a higher risk of HCC recurrence: HR 1.64 (95% CI 1.05–2.56), *p* = 0.03.

In PWH, the only independent predictors of HCC recurrence were advanced HCC and treatment with TACE, while a higher platelet count was associated with a lower risk of recurrence (Table S6). Independent predictors of HCC recurrence in PWOH are reported in Table S7.

## 4 Discussion

This study primarily compared HCC outcomes between PWH and PWOH. As one of the largest cohorts of PWH with HCC to date, our analysis provides an account of treatment approaches over an extended follow‐up period. Homogeneous data on the treatment strategies applied, encompassing not only initial therapies but also subsequent treatments, grant a level of detail that has been lacking in previous research.

Epidemiologically, in line with previous studies, our findings confirm that PWH were predominantly male, younger, and had more advanced liver disease at HCC diagnosis. They also exhibited higher rates of cirrhosis and thrombocytopenia compared to PWOH, supporting the hypothesis of accelerated fibrogenesis in HBV/HCV‐HIV coinfection [8, 29]. Although most patients in both groups presented with BCLC stage 0/A disease, a higher proportion of PWH had intermediate/advanced stage HCC, at diagnosis. The advanced stage at presentation may be explained by an increased oncogenic potential due to the synergistic effects of HIV and HBV/HCV. A recent report by Pinato et al. suggested that profound alterations in adaptive and innate immunity persist in PWH with HCC, despite ART‐induced viral suppression and preserved CD4+ T‐cell counts [12]. Other factors, including alcohol use or metabolic‐associated liver disease, may also have contributed to worse disease presentation in PWH. Although our study documented the primary aetiology of liver disease, we could not entirely exclude the coexistence of viral and metabolic or alcohol‐related liver disease in PWH. Despite the higher proportion of patients with advanced HCC at diagnosis, PWH with BCLC C HCC show a trend toward a lower tumour burden, likely due to the high adherence to the surveillance program.

The existing literature does not offer unequivocal evidence regarding differences in OS between PWH and non‐infected patients. In our study, despite PWH being diagnosed with more advanced HCC, even with comparable adherence to surveillance, the 5‐year OS was similar between the two groups. This was confirmed also considering only HCC‐related deaths and even after IPTW propensity score adjustment.

A previous large multicenter study has reported a 24% higher mortality risk for PWH with HCC compared to PWOH [21]. The poorer outcomes observed in this and other studies [6, 9, 13, 20] may be attributed to limited access to curative options such as LT, suboptimal ART and/or HBV/HCV therapy, or low CD4+ T‐cell counts—factors that have historically restricted treatment access and, consequently, survival [13, 18, 21]. Indeed, previous research has consistently shown increased mortality in PWH when access to curative treatments was limited [16, 18, 19, 22, 23]. Other possible contributors to higher mortality include tumour aggressiveness, as suggested by more advanced BCLC stages at diagnosis and higher recurrence rates [9, 25]. Our study comprehensively addressed these aspects by including a large number of cases evaluated over a prolonged period, with therapeutic strategies incorporating LT and sequential treatments. However, a possible effect of increased aggressiveness and reduced access to curative treatment on survival can be observed also in our study, as we observed an association between HIV infection and increased mortality risk within the first 12 months after HCC diagnosis. This finding can be partially attributed to the possible accelerated progression of cirrhosis and HCC already reported by other authors [8, 9, 12] and to the critical period during which downstaging strategies and curative treatments may have not been implemented yet. However, after IPTW propensity score adjustment, this excess of early mortality was no longer significant, hence we can't exclude bias due to the higher proportion of patients having advanced disease at baseline and to mitigating factors such as younger age or the introduction of direct‐acting antivirals for HCV treatment after 2014.

Conversely, the positive effect of access to curative treatments was clearer in our study when considering only patients with intermediate and advanced HCC. In BCLC B and BCLC C PWH the significant survival advantage could be explained by the lower tumour burden in patients with advanced disease at diagnosis, the higher access to curative treatment and the higher proportion of disease control achieved after first‐line treatment as compared to PWOH. In PWH we observed a shorter OS in patients with a lower CD4+ lymphocyte count, in line with previous reports. Considering the absence of significant differences in HCC stage, liver compensation and first‐line treatment, the difference in survival could be due to a higher immunosuppressive status not only in blood but also in the liver microenvironment, promoting HCC and cirrhosis progression [32].

The predictors of OS in the entire cohort and in PWH specifically were primarily related to age, underlying liver disease severity, HCC stage at diagnosis, and first‐line treatment. Importantly, no HIV‐specific factors independently influenced survival, likely reflecting the widespread use of effective ART and sustained HIV viral suppression. This finding is consistent with a study by Torgersen et al., which identified CD4+ T‐cell count as a predictor of OS only in patients without cirrhosis [33]. or recurrence risk in PWH. These results suggest that HCC treatment decisions in PWH should not be influenced by prior or current immunosuppression status. All therapeutic options, including LT, were available to both PWH and PWOH across the four participating centers. Unlike prior studies reporting higher rates of untreated HCC in PWH [9, 19], our findings indicate that 88% of PWH received at least one line of therapy. Interestingly, we also observed a higher proportion of curative treatment in PWH with advanced HCC, probably due to the lower tumour burden as compared to PWOH. Nevertheless, the rate of curative treatment at HCC diagnosis remained lower in PWH, mainly in early‐stage disease. This discrepancy may partly reflect differences in therapeutic approaches at different centers over time, particularly the increased use of TACE in BCLC 0/A disease. However, the overall rate of complete response did not differ significantly between groups. Among patients who achieved a complete response to initial HCC therapy, PWH experienced a shorter time to recurrence and higher recurrence rates after adjusting for baseline characteristics. This could be due to the higher aggressiveness of HCC in PWH as compared to PWOH, although we can't exclude a bias due to HCC stage in patients achieving complete response. The present study observed that, in patients with HCC who achieved a CR to first‐line treatment, seven (20%) were classified as BCLC C at the time of diagnosis, while no cases were observed in patients with PWH. Furthermore, no patients with advanced HCC achieved a complete response to treatment. As BCLC C patients have a higher risk of recurrence due to the presence of extrahepatic spread and/or macrovascular invasion, this could contribute to exacerbating the differences between the two cohorts.

However, these differences did not translate into worse OS, likely due to similar access to curative retreatment at recurrence in both groups. Furthermore, a higher proportion of PWH underwent LT during follow‐up, a therapeutic approach that has been shown to confer exceptional survival benefits in this population (OS rate of 90.7% at 2 years) [22].

This study has some limitations. Its retrospective design, high proportion of HCV RNA‐positive patients, and incomplete surveillance adherence data could introduce biases. Additionally, information on other liver damage factors was not always available. However, our cohort remains representative of real‐world clinical practice, as patients were managed at specialised referral centers. The inclusion of consecutive patients across all BCLC stages allowed for an evaluation of HIV's impact on both early and advanced HCC.

The patients in this study were monitored at one center for underlying liver disease, received antiviral therapy for HCV and HBV, and underwent six‐monthly surveillance, while at the other three centers, they were managed for both liver disease and HIV with antiretroviral treatment. This structured care in referral centers likely contributed to the higher surveillance rate as compared to the proportion reported in the literature (91%–88% vs. 52%) [34] and to the predominance of BCLC 0 and A stages at tumour diagnosis, underscoring the critical role of regular HCC surveillance.

The balancing of baseline characteristics through IPTW propensity score adjustment allows excluding the impact of other baseline characteristics; hence the similar survival in the two populations and the observed significant impact of HIV on recurrence should be considered reliable. Additionally, the inclusion of treatment data at recurrence provided a more comprehensive understanding of HCC outcomes in PWH.

In conclusion, despite PWH being diagnosed with more advanced HCC, their 5‐year survival remained comparable to that of PWOH. This was likely due to optimised HIV management, effective ART, and equitable access to curative HCC therapies, including early LT. These findings underscore the importance of ensuring rigorous HCC surveillance and timely access to optimal treatment for PWH under effective ART, ultimately improving long‐term outcomes in this population.

## Author Contributions

M.I. and A.S.o. conceived the study and wrote the article with E.A. and H.H.; E.A. made substantial contributions to the study design and analysis; B.M., F.P., A.S.o., A.S.i., M.F., M.B., M.M., and G.M. contributed to data collection and were actively involved in patient management; A.D.S. performed the analysis; and M.P., P.B., A.C., and P.L. critically revised the manuscript. All authors revised and approved the final version of the manuscript.

## Conflicts of Interest

M.I. participated in the advisory board and received speaker fees from Gilead Sciences, Bayer, AstraZeneca, Roche, Roche Diagnostics, EISAI, IPSEN, and MSD; E.A. received speaker fees from Roche and Gilead; A.S.o. received speaker fees from Abbvie and Gilead; P.L. participated in an advisory board and received speaker fees for AbbVie, Aligos, Altona, Antios, Eiger, Gilead Sciences, GlaxoSmithKline, Grifols, Janssen, MYR, Roboscreen, Roche Pharma/Diagnostics, and Vir.

## Supporting information


**Table S1:** Characteristics of patients after Inverse Probability Treatment Weighting (IPTW) propensity score.
**Table S2:** Univariable and multivariable Cox regression model to predict mortality in PWOH.
**Table S3:** First‐line HCC treatment allocation and response according to HIV status and BCLC stage.
**Table S4:** Treatment allocation according to HIV status and year of HCC diagnosis.
**Table S5:** Univariable and multivariable Cox regression model to predict HCC recurrence in 237 patients of the whole cohort with complete radiological response to first line treatments (132 failures).
**Table S6:** Univariable and multivariable Cox regression model to predict recurrence in 47 PWH with complete radiological response after first‐line treatment (28 events).
**Table S7:** Univariable and multivariable Cox regression model to predict recurrence in PWOH with complete radiological response after first‐line treatment.

**Figure S1:** Log–log survival plot assessing the proportional hazards assumption for HIV infection in the overall population. The progressive convergence of the curves suggests that the effect of HIV infection on mortality decreases over time, indicating non‐proportional hazards.

## Data Availability

The data that support the findings of this study are available from the corresponding author upon reasonable request.
