# Supplementary material for: Comparable 5‐Year Survival in People With and Without HIV Following Hepatocellular Carcinoma Diagnosis: A Multicenter Study
Source: Liver Int. 2025 Nov 18;45(12):e70437. doi: 10.1111/liv.70437 (PMC12625804; doi:10.1111/liv.70437)
Supplement: Supplementary file 1 — Table S1: Characteristics of patients after Inverse Probability Treatment Weighting (IPTW) propensity score. Table S2: Univariable and multivariable Cox regression model to predict mortality in PWOH. Table S3: First‐line HCC treatment allocation and response according to HIV status and BCLC stage. Table S4: Treatment allocation according to HIV status and year of HCC diagnosis. Table S5: Univariable and multivariable Cox regression model to predict HCC recurrence in 237 patients of the whole cohort with complete radiological response to first line treatments (132 failures). Table S6: Univariable and multivariable Cox regression model to predict recurrence in 47 PWH with complete radiological response after first‐line treatment (28 events). Table S7: Univariable and multivariable Cox regression model to predict recurrence in PWOH with complete radiological response after first‐line treatment. [file LIV-45-0-s001.docx]

Supplementary Table 1. Characteristics of patients after Inverse Probability Treatment Weighting (IPTW) propensity score

| **Characteristic** | **PWH group** | **PWOH** |
| --- | --- | --- |
| **Age, years*** | 57 (53-62) | 64 (55-72) |
| **Males, N (%)** | 45.1 (75) | 213.9 (72) |
| **BMI, Kg/m^2^*** | 23.8 (21.0-26.9) | 24.7 (22.8-27.4) |
| **Non viral** | 0 | 0 |
| **Diagnosis of cirrhosis, N (%)** | 59.8 (100%) | 294.2 (100%) |
| **CPT, N (%)**  **A**  **B**  **C** | 46.3 (78)  11 (18)  2.5 (4) | 229.2 (78)  52.5 (18)  12.5 (4) |
| **Creatinine, mg/dL*** | 1.0 (0.8-1.1) | 0.9 (0.7-1.1) |
| **Platelet count, 10^9^ cells/L*** | 121 (93-187) | 120 (88-162) |
| **AFP, ng/mL*** | 16.1 (5-145) | 15 (6-80) |
| **MVI and/or EHS, N (%)**  **None**  **MVI**  **EHS**  **Both** | 47.7 (80)  6.3 (10)  0.3 (1)  5.5 (9) | 252.8 (86)  20.3 (7)  5.8 (2)  15.3 (5) |
| **BCLC, N (%)**  **0**  **A**  **B**  **C**  **D** | 8.3 (14)  30.7 (51)  6.6 (11)  11 (19)  3.2 (5) | 55.4 (19)  144.6 (49)  38.0 (15)  12.5 (13)  4.2 (4) |

*Median (IQR). BMI: body mass index; CPT: Child-Pugh score; AFP: alpha-fetoprotein; MVI, macrovascular invasion; EHS, extra-hepatic spread; BCLC: Barcelona Clinic Liver Cancer.

Supplementary Table 2. Univariable and multivariable Cox regression model to predict mortality in PWOH

| **Variable** | **Type of variable** | **HR** | **95% CI** | **p-value** | **HR** | **95% CI** | **p-value** |
| --- | --- | --- | --- | --- | --- | --- | --- |
| **Males** | Yes vs no | 1.16 | 0.90-1.50 | 0.26 |  |  |  |
| **Age, years** | Continuous | 1.03 | 1.02-1.05 | <0.001 | 1.03 | 1.02-1.04 | <0.001 |
| **BMI** | Continuous | 0.98 | 0.96-1.01 | 0.28 |  |  |  |
| **Diabetes** | Yes vs no | 1.13 | 0.87-1.47 | 0.37 |  |  |  |
| **HCV** | Yes vs no | 0.91 | 0.73-1.13 | 0.39 |  |  |  |
| **HBV** | Yes vs no | 1.02 | 0.72-1.46 | 0.90 |  |  |  |
| **HBV+HDV** | Yes vs no | 0.93 | 0.46-1.87 | 0.83 |  |  |  |
| **HCV+HBV** | Yes vs no | 0.71 | 0.38-1.33 | 0.28 |  |  |  |
| **Non viral** | Yes vs no | 1.23 | 0.95-1.60 | 0.11 |  |  |  |
| **CPT**  **A**  **B**  **C** | vs A  vs A | 1 (ref)  1.35  11.98 | 1.04-1.75  5.74-25.00 | 0.02  <0.001 | 1 (ref)  1.16  1.20 | 0.87-1.56  0.14-10.68 | 0.30  0.87 |
| **ALBI score**  **1**  **2**  **3** | vs 1  vs 1 | 1 (ref)  1.18  2.40 | 0.95-1.47  1.18-4.90 | 0.14  0.02 | Omitted for collinearity | | |
| **MELD** | Continuous | 1.05 | 1.01-1.09 | 0.01 | Omitted for collinearity | | |
| **NLR** | Continuous | 1.15 | 0.96-1.36 | 0.12 |  |  |  |
| **Ascites** | Yes vs no | 1.27 | 1.01-1.59 | 0.04 | Omitted for collinearity | | |
| **HE** | Yes vs no | 1.51 | 1.07-2.11 | 0.02 | Omitted for collinearity | | |
| **E/G Varices** | Yes vs no | 1.35 | 1.09-1.69 | 0.007 | 1.39 | 1.09-1.78 | 0.009 |
| **Albumin, g/dL** | Continuous | 0.72 | 0.58-0.89 | 0.002 | Omitted for collinearity | | |
| **Bilirubin, mg/dL** | Continuous | 1.15 | 1.02-1.31 | 0.02 | Omitted for collinearity | | |
| **INR** | Continuous | 1.21 | 0.73-1.99 | 0.46 |  |  |  |
| **Creatinine, mg/dL** | Continuous | 1.63 | 1.09-2.42 | 0.02 | 1.31 | 0.86-1.99 | 0.20 |
| **Platelets, cells/mm3** | Continuous | 1.00 | 0.99-1.00 | 0.60 |  |  |  |
| **AFP>200 ng/mL** | Yes vs no | 2.57 | 1.92-3.43 | <0.001 | 1.68 | 1.18-2.40 | 0.004 |
| **“Milan Criteria” out** | Yes vs no | 2.63 | 2.10-3.28 | <0.001 | Omitted for collinearity | | |
| **Nodules categories**  **1 nodule**  **2-3 nodules**  **>3 nodules** | vs 1  vs 1 | 1 (ref)  1.47  3.10 | 1.16-1.88  2.22-4.19 | 0.002  <0.001 | Omitted for collinearity | | |
| **Maximum diameter, cm** | Continuous | 1.01 | 1.01-1.02 | <0.001 | Omitted for collinearity | | |
| **MVI, EHS or both**  **None**  **MVI**  **EHS**  **Both** | vs intrahepatic  vs intrahepatic  vs intrahepatic | 1 (ref)  5.67  4.16  15.04 | 3.72-8.65  2.31-7.50  7.73-29.27 | <0.001  <0.001  <0.001 | Omitted for collinearity | | |
| **BCLC**  **0**  **A**  **B**  **C**  **D** | vs 0  vs 0  vs 0  vs 0 | 1 (ref)  1.42  2.78  9.32  21.48 | 1.06-1.91  1.93-3.95  6.22-13.97  10.28-44.89 | 0.02  <0.001  <0.001  <0.001 | 1 (ref)  1.49  2.17  4.39  7.95 | 1.07-2.08  1.43-3.30  2.49-7.76  1.04-60.54 | 0.02  <0.001  <0.001  0.04 |
| **Treatment**  **LT**  **Resection**  **TA**  **TACE**  **TARE**  **Systemic treatment**  **BSC only** | Resection vs LT  TA vs LT  TACE vs LT  TARE vs LT  Systemic treatment vs LT  BSC vs LT | 1 (ref)  1.65  2.03  4.44  9.45  11.99  25.94 | 0.78-3.52  0.99-4.14  2.15-9.17  2.47-36.07  5.53-25.97  11.11-60.59 | 0.19  0.05  <0.001  0.001  <0.001  <0.001 | 1 (ref)  1.04  1.41  2.63  4.10  2.44  5.64 | 0.31-3.45  0.44-4.51  0.81-8.51  0.77-21.83  0.69-8.72  1.52-20.96 | 0.94  0.56  0.11  0.10  0.17  0.001 |

PWOH: people without HIV; HR: hazard ratio; CI: confidence interval; BMI: body mass index; HCV: hepatitis C; HBV: hepatitis B; HDV: hepatitis delta; CPT: Child-Pugh score; MELD: model for end-stage liver disease; ALBI score: albumin-bilirubin score; HE: hepatic encephalopathy; E/G: Esophagogastric; INR: international normalized ratio; NLR: neutrophil/lymphocyte ratio; AFP: alpha-fetoprotein; MVI, macrovascular invasion; EHS, extra-hepatic spread; BCLC: Barcelona Clinic Liver Cancer; LT: liver transplant; TA: thermal ablation; TACE: transarterial chemoembolization; TARE: transarterial radioembolization; BSC: best supportive care, ref: reference.

Supplementary Table 3. First-line HCC treatment allocation and response according to HIV status and BCLC stage.

|  | **BCLC 0** | | | **BCLC A** | | | **BCLC B** | | | **BCLC C** | | | **BCLC D** | | |
| --- | --- | --- | --- | --- | --- | --- | --- | --- | --- | --- | --- | --- | --- | --- | --- |
| **First-line treatments** | **PWH**  **(n=21)** | **PWOH**  **(N=97)** | **p-value** | **PWH**  **(N=59)** | **PWOH**  **(N=226)** | **p-value** | **PWH**  **(n=16)** | **PWOH**  **(N=82)** | **p-value** | **PWH**  **(n=31)** | **PWOH**  **(N=48)** | **p-value** | **PWH**  **(n=16)** | **PWOH**  **(N=10)** | **p-value** |
| Treatment  LT  Resection  TA  Resection+TA  TA+TACE  TACE  TACE+TKI  TARE  Systemic treatment, TKI  Systemic treatment, IO-based  BSC only | 2 (9.5%)  5 (24%)  12 (57%)  0  0  2 (9.5%)  0  0  0  0  0 | 6 (6.2%)  9 (9.3%)  66 (68%)  0  0  14 (14.4%)  0  0  0  0  2 (2.1%) | 0.24 | 7 (12%)  16 (27%)  12 (20%)  0  0  20 (34%)  0  1 (2%)  2 (3%)  0  1 (2%) | 8 (3.5%)  60 (26.5%)  91 (40%)  2 (1%)  10 (4%)  49 (22%)  1 (0.5%)  0  1 (0.5%)  2 (1%)  2 (1%) | 0.002 | 0  3 (19%)  0  0  0  9 (56%)  0  3 (19%)  0  0  1 (6%) | 2 (2%)  6 (8%)  12 (15%)  2 (2%)  2 (2%)  42 (52%)  2 (2%)  3 (4%)  6 (7%)  1 (1%)  4 (5%) | 0.19 | 1 (3%)  8 (26%)  3 (10%)  0  0  6 (20%)  1 (3%)  1 (3%)  5 (16%)  1 (3%)  5 (16%) | 0  0  3 (6%)  1 (2%)  0  4 (9%)  0  2 (4%)  30 (62%)  1 (2%)  7 (15%) | <0.001 | 9  0  0  0  0  1 (6%)  0  0  5 (31%)  0  10 (63%) | 0  0  0  0  0  3 (30%)  0  0  1 (10%)  0  6 (60%) | 0.20 |
| Curative treatments* | 18 (90%) | 81 (83%) | 0.46 | 35 (59%) | 171 (76%) | 0.01 | 3 (19%) | 24 (29%) | 0.34 | 12 (38%) | 4 (8%) | 0.002 | 1 (11%) | 0 | 1.00 |
| Response to 1st line treatment  Complete response  Partial response  Stable disease  Progression | 13 (62%)  6 (28%)  1 (5%)  1 (5%) | 66 (68%)  17 (18%)  0  14 (14%) | 0.07 | 31 (53%)  14 (24%)  9 (15%)  5 (8%) | 122 (54%)  55 (24%)  4 (2%)  45 (20%) | <0.001 | 5 (31%)  5 (31%)  4 (25%)  2 (13%) | 18 (22%)  31 (38%)  1 (1%)  32 (39%) | <0.001 | 7 (22%)  8 (26%)  3 (10%)  13 (42%) | 0  8 (17%)  2 (4%)  38 (79%) | 0.001 | 0  0  2 (12%)  14 (88%) | 0  0  1 (10%)  9 (90%) | 0.84 |

* Curative treatments included liver transplantation, surgical resection, and radiofrequency or microwave thermal ablation. PWH, people with HIV; PWOH, people without HIV; BCLC, Barcelona Clinic Liver Cancer; LT, liver transplantation; TA, thermal ablation; TACE, transarterial chemoembolization; TARE, transarterial radioembolization; TKI, tyrosine kinase inhibitors; IO, immunotherapy; BSC, best supportive care.

Supplementary Table 4. Treatment allocation according to HIV status and year of HCC diagnosis

| **PWH** | | | | |
| --- | --- | --- | --- | --- |
| **Treatments** | **Cohort 1ˣ**  **(n=29)** | **Cohort 2 ˣ**  **(N=71)** | **Cohort 3 ˣ**  **(N=43)** | **p-value** |
| 1^st^ line treatment, N (%)  LT  Resection  TA  TACE  TACE+TKI  TARE  Systemic treatment, TKI  Systemic treatment, IO based  BSC | 1 (4)  8 (28)  5 (17)  5 (17)  0  0  3 (10)  0  7 (24) | 6 (9)  12 (17)  15 (21)  18 (25)  1 (1)  5 (7)  8 (11)  0  6 (9) | 3 (7)  12 (18)  7 (16)  15 (35)  0  0  1 (2)  1 (2)  4 (10) | 0.21 |
| Curative treatments*, N (%) | 14 (48) | 33 (47) | 22 (51) | 0.89 |
| LT in first line, N (%) | 1 (4) | 6 (9) | 3 (7) | 0.67 |
| LT any time during follow-up, N (%) | 3 (10) | 22 (31) | 9 (21) | 0.08 |
| **PWOH** | | | | |
| 1^st^ line treatment, N (%)  LT  Resection  TA  Resection+TA  TA+TACE  TACE  TACE+TKI  TARE  Systemic treatment, TKI  Systemic treatment, IO based  BSC | 13 (4)  49 (15)  132 (39.7)  3 (1)  8 (2)  83 (25)  3 (1)  1 (0.3)  28 (8)  0  12 (4) | 1 (3)  4 (10)  13 (33)  0  3 (8)  12 (31)  0  0  4 (10)  0  2 (5) | 2 (2)  22 (24)  27 (29)  2 (2)  1 (1)  17 (19)  0  4 (4)  6 (7)  4 (4)  7 (8) | <0.001 |
| Curative treatments*, N (%) | 205 (62) | 21 (54) | 54 (57) | 0.59 |
| LT in first line, N (%) | 13 (4) | 1 (3) | 2 (2) | 0.69 |
| LT any time during follow-up, N (%) | 35 (10) | 5 (13) | 12 (13) | 0.08 |

ˣ Cohort 1: HCC diagnosis from 2005 to 2010; Cohort 2: HCC diagnosis from 2011 to 2015; Cohort 3: diagnosis from 2016 to 2023

*Curative treatments included liver transplantation, surgical resection, and radiofrequency or microwave thermal ablation. PWH, people with HIV; PWOH, people without HIV; LT, liver transplantation; TA, thermal ablation; TACE, transarterial chemoembolization; TARE, transarterial radioembolization; TKI, tyrosine kinase inhibitor; IO, immunotherapy; BSC, best supportive care

Supplementary Table 5. Univariable and multivariable Cox regression model to predict HCC recurrence in 237 patients of the whole cohort with complete radiological response to first line treatments (132 failures).

| **Variable** | **Type of variable** | **HR** | **95% CI** | **p-value** | **HR** | **95% CI** | **p-value** |
| --- | --- | --- | --- | --- | --- | --- | --- |
| Born males | Yes vs no | 1.17 | 0.80-1.71 | 0.43 |  |  |  |
| Age, years | Continuous | 0.97 | 0.65-1.45 | 0.89 |  |  |  |
| BMI, Kg/m^2^ | Continuous | 0.97 | 0.93-1.01 | 0.14 |  |  |  |
| Diabetes | Yes vs no | 1.12 | 0.74-1.71 | 0.59 |  |  |  |
| HCV | Yes vs no | 1.06 | 0.75-1.50 | 0.74 |  |  |  |
| HBV | Yes vs no | 0.67 | 0.34-1.31 | 0.24 |  |  |  |
| HBV+HDV | Yes vs no | 0.72 | 0.29-1.77 | 0.48 |  |  |  |
| HCV+HBV | Yes vs no | 1.65 | 0.89-3.05 | 0.11 |  |  |  |
| Non viral | Yes vs no | 0.99 | 0.64-1.54 | 0.97 |  |  |  |
| HIV infection | Yes vs no | 1.10 | 0.74-1.62 | 0.65 |  |  |  |
| Cirrhosis | Yes vs no | 1.11 | 0.54-2.27 | 0.77 |  |  |  |
| CPT  A  B  C | vs A  vs A | 1 (reference)  0.94  NA | 0.61-1.45  NA | 0.78  NA |  |  |  |
| ALBI score  1  2  3 | vs 1  vs 1 | 1 (reference)  1.00  NA | 0.72-1.40  NA | 0.99  NA |  |  |  |
| MELD | Continuous | 0.97 | 0.91-1.03 | 0.39 |  |  |  |
| NLR | Continuous | 1.09 | 0.87-1.37 | 0.45 |  |  |  |
| Ascites | Yes vs no | 0.79 | 0.52-1.18 | 0.25 |  |  |  |
| HE | Yes vs no | 1.74 | 1.02-2.99 | 0.04 | 2.13 | 1.20-3.75 | 0.009 |
| E/G varices | Yes vs no | 0.83 | 0.57-1.21 | 0.34 |  |  |  |
| Albumin, g/dL | Continuous | 1.20 | 0.85-1.69 | 0.29 |  |  |  |
| Bilirubin, mg/dL | Continuous | 0.79 | 0.18-1.16 | 0.08 |  |  |  |
| INR | Continuous | 0.79 | 0.46-1.36 | 0.39 |  |  |  |
| Creatinine, mg/dL | Continuous | 2.08 | 1.18-3.65 | 0.01 | 2.54 | 1.34-4.83 | 0.004 |
| Platelets, cells/mm3 | Continuous | 1.14 | 0.81-1.62 | 0.45 |  |  |  |
| AFP>200 ng/mL | Yes vs no | 1.13 | 0.62-2.05 | 0.68 |  |  |  |
| BCLC  0  A  B  C | vs 0  vs 0  vs 0 | 1 (reference)  1.26  1.81  3.06 | 0.62-1.37  0.99-3.27  1.20-7.77 | 0.68  0.05  0.02 | 1.31  1.89  2.09 | 0.86-1.99  0.99-3.60  0.63-6.96 | 0.21  0.05  0.23 |
| Nodules categories  1  2-3  >3 | vs 1  vs 1 | 1 (reference)  1.53  0.74 | 1.06-2.06  0.18-3.02 | 0.03  0.68 | Omitted for collinearity | | |
| Maximum diameter, cm | Continuous | 1.01 | 0.99-1.01 | 0.12 |  |  |  |
| MVI, EHS or both  MVI  EHS  Both | vs intrahepatic  vs intrahepatic  vs intrahepatic | 5.45  NA  NA | 1.98-15.02  NA  NA | 0.001  NA  NA | Omitted for collinearity | | |
| “Milan Criteria” out | Yes vs no | 1.40 | 0.92-2.12 | 0.12 |  |  |  |
| Treatment  LT  Resection  TA  TACE | vs LT  vs LT  vs LT | 1 (reference)  9.27  9.60  14.96 | 2.88-29.79  3.02-30.58  4.39-50.99 | <0.001  <0.001  <0.001 | 7.60  9.78  13.45 | 2.32-24.82  3.03-31.52  3.87-46.77 | 0.001  <0.001  <0.001 |

BMI, body mass index; HCV, hepatitis C; HBV, hepatitis B; HDV, hepatitis delta; HIV, human immunodeficiency virus; CPT, Child-Pugh score; MELD, model for end-stage liver disease; ALBI score, albumin-bilirubin score; HE, hepatic encephalopathy; E/G: Esophagogastric; NLR, neutrophil/lymphocyte ratio; AFP, alpha-fetoprotein; MVI, macrovascular invasion; EHS, extrahepatic spread; MC: Milan Criteria; BCLC: Barcelona Clinic Liver Cancer; LT, liver transplant; TA, thermal ablation; TACE, transarterial chemoembolization.

Supplementary Table 6. Univariable and multivariable Cox regression model to predict recurrence in 47 PWH with complete radiological response after first-line treatment (28 events).

| **Variable** | **Type of variable** | **HR** | **95% CI** | **p-value** | **HR** | 95% CI | p-value |
| --- | --- | --- | --- | --- | --- | --- | --- |
| Born males | Yes vs no | 0.77 | 0.29-2.03 | 0.59 |  |  |  |
| Age, years | Continuous | 1.01 | 0.94-1.08 | 0.82 |  |  |  |
| BMI | Continuous | 0.97 | 0.87-1.08 | 0.58 |  |  |  |
| Diabetes | Yes vs no | 1.13 | 0.48-2.67 | 0.77 |  |  |  |
| HCV | Yes vs no | 0.89 | 0.42-1.86 | 0.75 |  |  |  |
| HBV | Yes vs no | 0.42 | 0.06-3.07 | 0.39 |  |  |  |
| HBV+HDV | Yes vs no | 0.82 | 0.25-2.71 | 0.75 |  |  |  |
| HCV+HBV | Yes vs no | 1.87 | 0.78-4.45 | 0.16 |  |  |  |
| Non-viral | Yes vs no | NA | NA | NA |  |  |  |
| CPT  A  B  C | vs A  vs A | 1 (ref)  1.41  NA | 2.07-5.67  NA | 0.57  NA |  |  |  |
| ALBI  1  2  3 | vs 1  vs 1 | 1 (ref)  1.72  NA | 0.75-3.99  0.30-3.28 | 0.20  NA |  |  |  |
| MELD | Continuous | 0.98 | 0.62-1.49 | 0.72 |  |  |  |
| NLR | Continuous | 0.96 | 0.93-1.34 | 0.86 |  |  |  |
| Ascites | Yes vs no | 0.35 | 0.08-1.48 | 0.15 |  |  |  |
| HE | Yes vs no | 0.65 | 0.09-4.83 | 0.68 |  |  |  |
| E/G varices | Yes vs no | 0.53 | 0.18-1.55 | 0.25 |  |  |  |
| AIDS | Yes vs no | 1.33 | 0.54-3.31 | 0.53 |  |  |  |
| CD4 count  ≥200 cells/mm3  <200 cells/mm3 | vs ≥200 | 1 (ref)  2.66 | 0.95-7-52 | 0.06 |  |  |  |
| HIV RNA positive | Yes vs no | 2.57 | 0.58-11.32 | 0.21 |  |  |  |
| Albumin, g/dL | Continuous | 0.81 | 0.42-1.54 | 0.51 |  |  |  |
| Bilirubin, mg/dL | Continuous | 0.81 | 0.42-1.54 | 0.52 |  |  |  |
| INR | Continuous | 1.00 | 0.54-1.84 | 0.99 |  |  |  |
| Creatinine, mg/dL | Continuous | 0.35 | 0.05-2.65 | 0.31 |  |  |  |
| Platelets, cells/mm3 | Continuous | 0.99 | 0.98-0.99 | 0.02 | 0.99 | 0.98-0.99 | 0.01 |
| AFP>200 ng/mL | Yes vs no | 1.46 | 0.50-4.36 | 0.50 |  |  |  |
| “Milan Criteria” out | Yes vs no | 1.90 | 0.91-3.96 | 0.09 |  |  |  |
| Nodules categories  1 nodule  2-3 nodules | vs single | 1 (ref)  1.90 | 0.91-3.96 | 0.08 |  |  |  |
| Maximum diameter, cm | Continuous | 1.01 | 0.99-1.03 | 0.38 |  |  |  |
| MVI, EHS or both  None  MVI  EHS  both | vs intrahepatic  vs intrahepatic  vs intrahepatic | 4.56  NA  NA | 1.49-13.94  NA  NA | 0.008  NA  NA | Omitted for collinearity | | |
| BCLC  0  A  B  C | vs 0  vs 0  vs 0 | 1 (ref)  1.45  2.62  3.63 | 0.59-3.54  0.66-10.32  1.11-11.94 | 0.41  0.17  0.03 | 1 (ref)  1.79  3.39  4.48 | 0.53-6.00  0.67-17.10  1.07-18.69 | 0.35  0.14  0.04 |
| Treatment  LT  Resection  TA  TACE | vs LT  vs LT  vs LT | 1 (ref)  4.91  5.45  10.62 | 1.07-22.47  1.15-25.82  2.15-52.44 | 0.04  0.03  0.004 | 1 (ref)  4.43  5.47  7.42 | 0.74-22.63  0.82-34.08  1.17-38.52 | 0.11  0.08  0.03 |

HR: hazard ratio; CI: confidence interval; BMI: body mass index; HCV: hepatitis C; HBV: hepatitis B; HDV: hepatitis delta; HIV: human immunodeficiency virus; CPT: Child-Pugh score; MELD: model for end-stage liver disease; ALBI score: albumin-bilirubin score; HE: hepatic encephalopathy; E/G: Esophagogastric; AIDS: acquired immunodeficiency syndrome; CD: cluster of differentiation; NLR: neutrophil/lymphocyte ratio; AFP: alpha-fetoprotein; MVI, macrovascular invasion; EHS, extra-hepatic spread; BCLC: Barcelona Clinic Liver Cancer; LT: liver transplant; TA: thermal ablation; TACE: transarterial chemoembolization; ref: reference

Supplementary Table 7. Univariable and multivariable Cox regression model to predict recurrence in PWOH with complete radiological response after first-line treatment.

| **Variable** | **Type of variable** | **HR** | **95% CI** | **p-value** | **HR** | **95% CI** | **p-value** |
| --- | --- | --- | --- | --- | --- | --- | --- |
| **Males** | Yes vs no | 1.27 | 0.83-1.94 | 0.27 |  |  |  |
| **Age, years** | Continuous | 1.03 | 0.99-1.04 | 0.06 |  |  |  |
| **BMI, kg/m^2^** | Continuous | 0.98 | 0.93-1.02 | 0.31 |  |  |  |
| **Diabetes** | Yes vs no | 0.93 | 0.59-1.47 | 0.77 |  |  |  |
| **HCV** | Yes vs no | 1.11 | 0.75-1.64 | 0.60 |  |  |  |
| **HBV** | Yes vs no | 0.74 | 0.36-1.52 | 0.41 |  |  |  |
| **HBV+HDV** | Yes vs no | 0.61 | 0.15-2.46 | 0.48 |  |  |  |
| **HCV+HBV** | Yes vs no | 1.22 | 0.45-3.29 | 0.70 |  |  |  |
| **Non viral** | Yes vs no | 1.02 | 0.65-1.60 | 0.92 |  |  |  |
| **Cirrhosis** | Yes vs no | 1.19 | 0.55-2.56 | 0.65 |  |  |  |
| **CPT**  **A**  **B** | vs B | 1 (ref)  0.89 | 0.55-1.44 | 0.64 |  |  |  |
| **ALBI score**  **1**  **2**  **3** | vs 1  vs 1 | 1 (ref)  0.86  NA | 0.56-1.26  NA | 0.45  NA |  |  |  |
| **MELD** | Continuous | 0.97 | 0.90-1.05 | 0.45 |  |  |  |
| **NLR** | Continuous | 1.13 | 0.84-1.51 | 0.42 |  |  |  |
| **Ascites** | Yes vs no | 0.88 | 0.57-1.36 | 0.58 |  |  |  |
| **HE** | Yes vs no | 2.10 | 1.19-3.71 | 0.01 | 2.24 | 1.24-4.04 | 0.007 |
| **E/G Varices** | Yes vs no | 0.90 | 0.60-1.37 | 0.63 |  |  |  |
| **Albumin, g/dL** | Continuous | 1.41 | 0.95-2.10 | 0.09 |  |  |  |
| **Bilirubin, mg/dL** | Continuous | 0.79 | 0.59-1.06 | 0.11 |  |  |  |
| **INR** | Continuous | 0.54 | 0.23-1.28 | 0.16 |  |  |  |
| **Creatinine, mg/dL** | Continuous | 2.97 | 1.51-5.87 | 0.002 | 2.72 | 1.36-5.42 | 0.005 |
| **Platelets, cells/mm3** | Continuous | 1.00 | 0.99-1.00 | 0.99 |  |  |  |
| **AFP>200 ng/mL** | Yes vs no | 0.98 | 0.48-2.01 | 0.96 |  |  |  |
| **BCLC**  **0**  **A**  **B** | vs 0  vs 0 | 1 (ref)  1.21  1.69 | 0.81-1.83  0.87-3.27 | 0.19 |  |  |  |
| **Nodules categories**  **1**  **2-3**  **>3** | vs 1  vs 1 | 1 (ref)  1.43  0.74 | 0.93-2.18  0.18-3.01 | 0.10  0.67 |  |  |  |
| **Maximum diameter, cm** | Continuous | 1.01 | 0.99-1.01 | 0.14 |  |  |  |
| **“Milan Criteria” out** | Yes vs no | 1.19 | 0.73-1.95 | 0.48 |  |  |  |
| **Treatment**  **LT**  **Resection**  **TA**  **TACE** | vs LT  vs LT  vs LT | 17.71  18.41  22.60 | 2.43-129.20  2.55-133.05  2.88-177.35 | 0.005  0.004  0.003 | 16.05  18.52  23.49 | 2.19-117.42  2.56-134.09  2.99-184.46 | 0.006  0.004  0.003 |

PWOH: people without HIV; HR: hazard ratio; CI: confidence interval; BMI: body mass index; HCV: hepatitis C; HBV: hepatitis B; HDV: hepatitis delta; CPT: Child-Pugh score; MELD: model for end-stage liver disease; ALBI score: albumin-bilirubin score; HE: hepatic encephalopathy; E/G: Esophagogastric; INR: international normalized ratio; NLR: neutrophil/lymphocyte ratio; AFP: alpha-fetoprotein; MVI, macrovascular invasion; EHS, extra-hepatic spread; BCLC: Barcelona Clinic Liver Cancer; LT: liver transplant; TA: thermal ablation; TACE: transarterial chemoembolization; ref: reference.
