# Supplementary figures and images for: Comparable 5‐Year Survival in People With and Without HIV Following Hepatocellular Carcinoma Diagnosis: A Multicenter Study
Source: Liver Int. 2025 Nov 18;45(12):e70437. doi: 10.1111/liv.70437 (PMC12625804; doi:10.1111/liv.70437)

## Slide 1
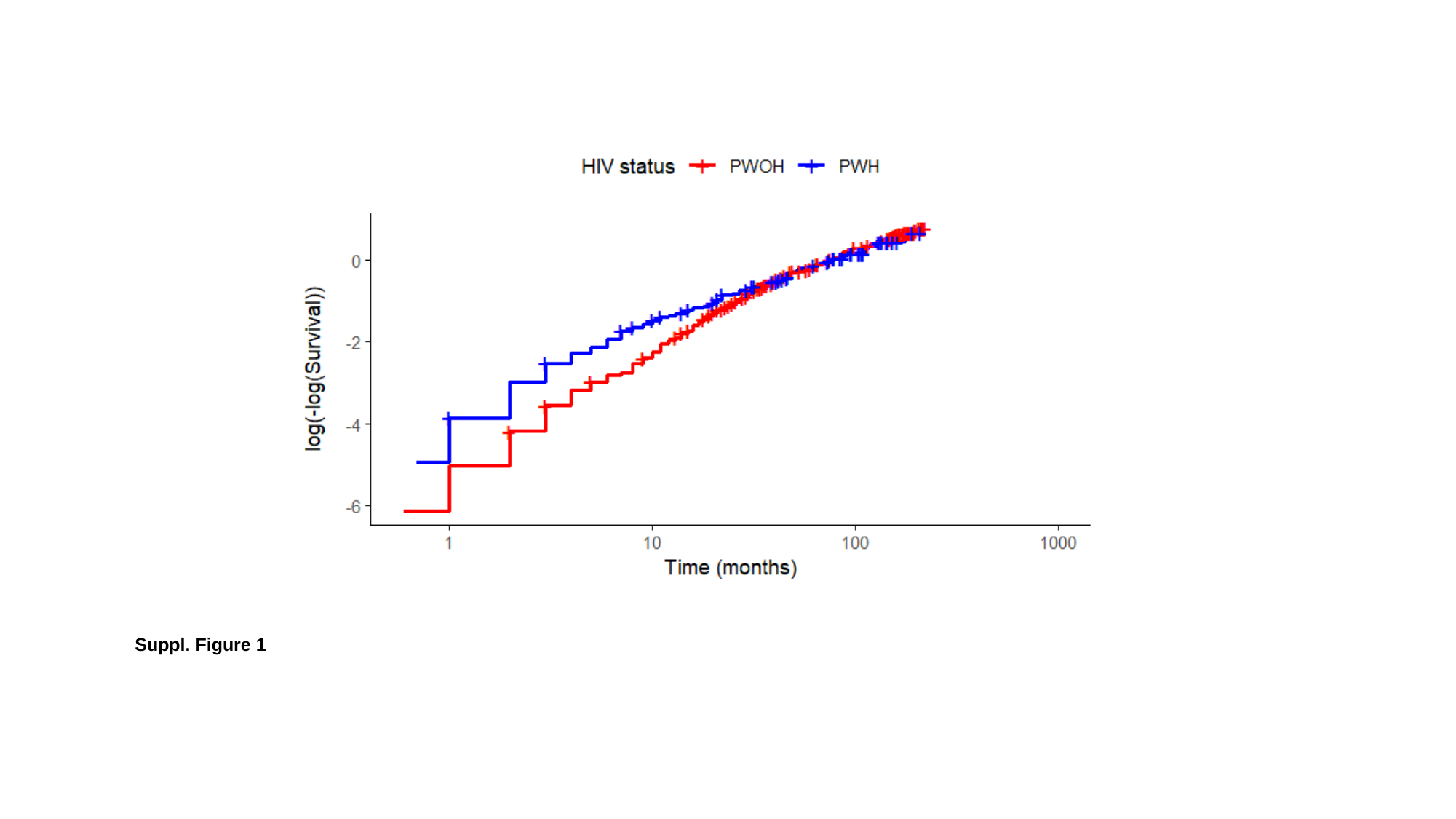

Suppl. Figure 1

Supplement: Supplementary file 2 — Figure S1: Log–log survival plot assessing the proportional hazards assumption for HIV infection in the overall population. The progressive convergence of the curves suggests that the effect of HIV infection on mortality decreases over time, indicating non‐proportional hazards. [file LIV-45-0-s002.pptx]
